# Breaking the ice: uncovering miPEP171b control of grapevine cold stress response

**DOI:** 10.1093/plcell/koag326

**Published:** 2026-10-27

**Authors:** Soledad Traubenik, Francisco Sánchez-Rodríguez

**Affiliations:** Assistant Features Editor, The Plant Cell, American Society of Plant Biologists; Institute of Plant Sciences Paris-Saclay (IPS2), CNRS, INRAE, Université Paris-Cité, Gif-sur-Yvette 91190, France; Instituto de Hortofruticultura Subtropical y Mediterránea “La Mayora” (IHSM “La Mayora”), Universidad de Málaga–Consejo Superior de Investigaciones Científicas (UMA-CSIC), Campus Teatinos, Málaga 29010, Spain

MicroRNAs (miRNAs) are small regulatory RNAs that control numerous biological processes, including plant development and responses to environmental cues. They are produced from primary miRNA transcripts (pri-miRNAs) and regulate gene expression through sequence complementarity, leading to target transcript cleavage or translational inhibition (Song et al. 2019). Although widely considered non-coding RNAs, pri-miRNAs may contain short open reading frames encoding regulatory peptides (miPEPs) which can establish a positive feedback loop by increasing the accumulation of the associated miRNA (Lauressergues et al. 2015). The importance of miPEPs in plant adaptation has also been shown in crops. In rice, miPEP156e enhances miR156 accumulation and represses its SQUAMOSA PROMOTER BINDING PROTEIN-LIKE (SPL) targets, improving cadmium tolerance (Lu et al. 2024), while miPEP172b increases miR172b accumulation and represses its target INDETERMINATE SPIKELET1 (IDS1), enhancing salt tolerance (Lu et al. 2025). Despite these promising examples, our understanding of the diversity and relevance of miPEPs in crop stress responses is still developing. Expanding their characterization across crop species and environmental conditions will help establish their broader relevance in plant stress adaptation.

Grapevine (*Vitis vinifera L.*), one of the most widely cultivated fruit crops worldwide, is particularly vulnerable to cold stress, with most cultivated Eurasian varieties showing poor cold tolerance and susceptibility to frost damage. Fan and colleagues (2026) have now identified a cold-responsive miPEP as a regulator of the grapevine cold stress response, uncovering a miPEP-driven regulatory module linking miRNA regulation to the epigenetic control of cold-responsive genes. Combining biochemical, genetic, and multi-omics approaches, they traced the pathway from a single miPEP to the epigenetic control of cold-responsive genes. To identify miPEPs involved in the grapevine cold response, the authors performed liquid chromatography with tandem mass spectrometry (LC-MS/MS)-based quantitative proteomics in cold-treated plants. Among those identified, miPEP171b emerged as a prominent cold-responsive peptide, showing high expression levels and a marked increase upon cold exposure. Reporter assays further confirmed that its open reading frame is actively translated in planta. Exogenous application of the synthetic miPEP171b increased vvi-miR171b accumulation, enhanced cold tolerance, and downregulated 2 known miR171 target genes, supporting a positive feedback mechanism with its cognate miRNA.

This raises the question: does miPEP171b enhance cold tolerance through its cognate miRNA, vvi-miR171b? In vvi-miR171b knockout calli, exogenous miPEP171b largely lost its protective effect, indicating that the peptide acts primarily through vvi-miR171b. Consistently, vvi-miR171b knockdown or knockout increased cold sensitivity, whereas its overexpression decreased it—establishing vvi-miR171b as a positive regulator of the cold response.

And how does vvi-miR171b mediate its effect on cold tolerance? The answer appears to lie in its downstream target, the SCARECROW-LIKE transcription factor VvSCL15 (Chen et al. 2020). Fan and colleagues showed that VvSCL15 acts as a negative regulator of cold tolerance: its expression declines under cold stress, whereas its knockdown enhances cold tolerance. Mechanistically, this effect involves histone deacetylation. Building on the known interaction between SCL15 and HDA19 in Arabidopsis (Gao et al. 2015), they demonstrated that VvSCL15 interacts with the grapevine histone deacetylase VvHDA19, thereby linking miRNA regulation to chromatin modification. VvSCL15 overexpression reduced H3 acetylation and the expression of cold-responsive genes. Importantly, VvSCL15 bound the promoters of several of these genes, where it reduced H3K9 acetylation and suppressed their transcription (Fig. 1).

**Figure 1:**
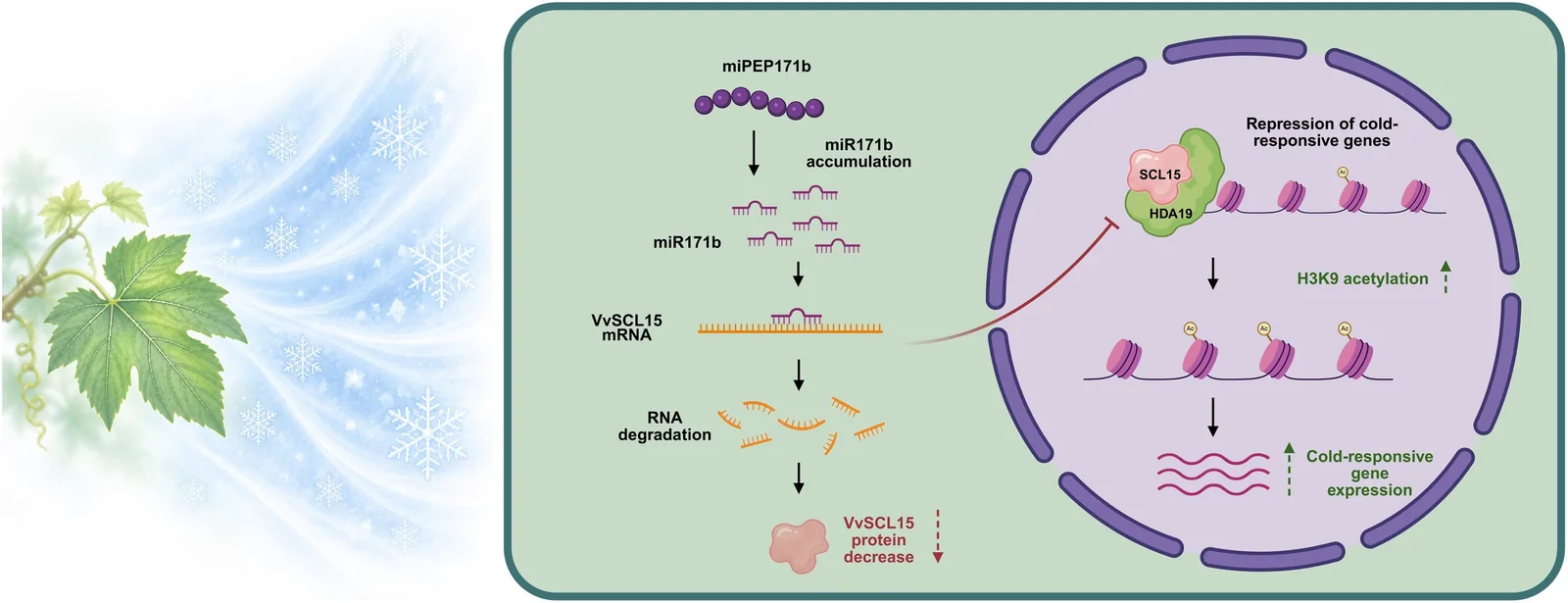
The miPEP171b-miR171b-VvSCL15/VvHDA19 module regulates the grapevine cold response. Cold-induced miPEP171b increases vvi-miR171b accumulation, promoting VvSCL15 mRNA degradation and reducing VvSCL15 protein levels. Reduced VvSCL15 limits VvSCL15-VvHDA19-mediated repression, leading to increased H3K9 acetylation at cold-responsive genes and enhanced gene expression. Created using BioRender.com. Leaf and cold wind illustrations generated using AI.

By assembling this regulatory pathway piece by piece, the authors connect a small peptide to the epigenetic control of cold-responsive genes. In doing so, they add a new dimension to miPEP biology: beyond establishing miPEP171b as a positive regulator of cold tolerance, this work provides a mechanistic understanding of how an exogenously applied miPEP exerts its effects in an agronomically important crop, strengthening the potential of miPEPs as non-transgenic tools for improving crop resilience.

## Recent related articles in *The Plant Cell*

**Dong et al., 2025** identifies wheat plant elicitor peptides (TaPeps) that activate immune signaling through TaPEPR1 and enhance resistance to Fusarium head blight, highlighting the potential of exogenously applied peptides for crop protection.

**Sun et al., 2024** reveals how the histone deacetylase OsHDA716 represses chilling tolerance in rice by deacetylating the transcription factor OsbZIP46, linking protein deacetylation to the regulation of cold-responsive genes.

## Data Availability

No datasets were generated or analysed.
